# Physics and semantic informed multi-sensor calibration via optimization theory and self-supervised learning

**DOI:** 10.1038/s41598-024-53009-z

**Published:** 2024-01-30

**Authors:** Shmuel Y. Hayoun, Meir Halachmi, Doron Serebro, Kfir Twizer, Elinor Medezinski, Liron Korkidi, Moshik Cohen, Itai Orr

**Affiliations:** 1 Wisense Technologies Ltd., Tel Aviv, Israel; 2 Department of Computer Science, Technion-Israel Institute of Technology, Haifa, Israel

**Keywords:** Computational science, Information technology, Electrical and electronic engineering

## Abstract

Widespread adaptation of autonomous, robotic systems relies greatly on safe and reliable operation, which in many cases is derived from the ability to maintain accurate and robust perception capabilities. Environmental and operational conditions as well as improper maintenance can produce calibration errors inhibiting sensor fusion and, consequently, degrading the perception performance and overall system usability. Traditionally, sensor calibration is performed in a controlled environment with one or more known targets. Such a procedure can only be carried out in between operations and is done manually; a tedious task if it must be conducted on a regular basis. This creates an acute need for online targetless methods, capable of yielding a set of geometric transformations based on perceived environmental features. However, the often-required redundancy in sensing modalities poses further challenges, as the features captured by each sensor and their distinctiveness may vary. We present a holistic approach to performing joint calibration of a camera–lidar–radar trio in a representative autonomous driving application. Leveraging prior knowledge and physical properties of these sensing modalities together with semantic information, we propose two targetless calibration methods within a cost minimization framework: the first via direct online optimization, and the second through self-supervised learning (SSL).

## Introduction

**Figure 1:**
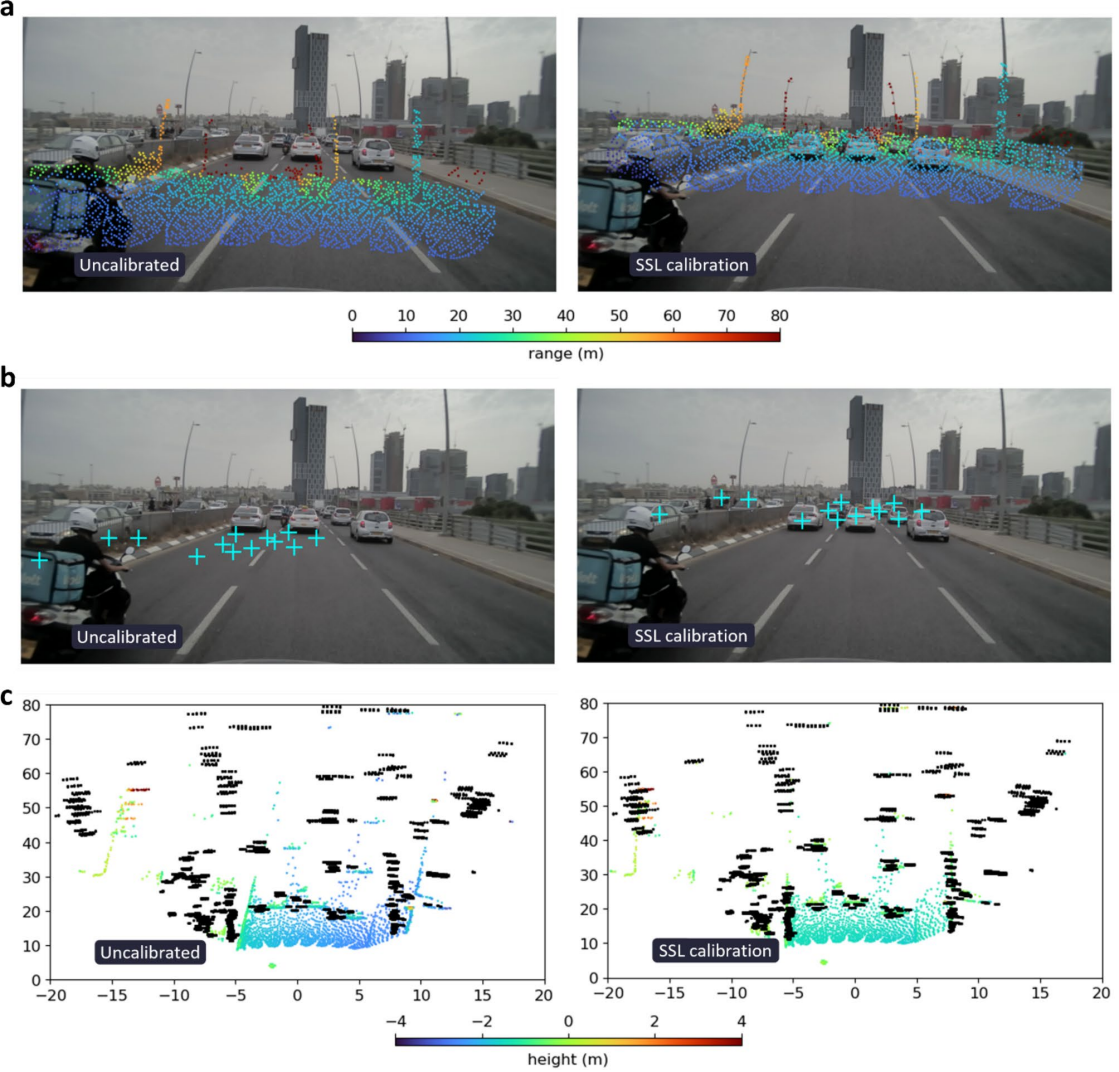
Physics and semantic informed calibration. Front views of the sensors’ spatial measurements (**a**, **b**) and a top view of the lidar and radar point clouds (**c**). Each view is shown in its uncalibrated state (left) and calibrated state, resulting from our proposed approach (right). In (**a**) the camera–lidar calibration result is illustrated; the projected lidar point cloud (colored by range) is clearly misaligned in the camera image to the left, whereas on the right our method achieves a good calibration across the entire scene. In (**b**) the camera–radar calibration is shown; the radar, represented by dynamic track detections (cyan ‘+’ markers), that are misaligned with the moving vehicles in the image on the left, are visibly well aligned on the right. In (**c**) a bird’s-eye view of the uncalibrated and calibrated lidar (colored by height) and radar (black points) is given with axes units in meters; on the right the alignment is discernible from the overlapping detections of objects such as vehicles and the guard rails, unlike the severe misalignment seen on the left.

Recent years have witnessed the emergence of numerous outdoor-based autonomous systems in a variety of fields, such as distribution^1^, agriculture^2^, search and rescue^3^, transportation^4^ and more. Their ability to accurately perceive the environment in diverse settings is necessary for versatile and robust operation. With the increasing level of these systems’ autonomy the idea of sensor fusion to improve perception capabilities^5–10^ has gained much attention and is currently a major research topic. Understandably, the effectiveness of the fusion process is correlated to the level of correspondence between multiple measurements of the same object. Therefore, to maintain accurate perception over time, one must retain precisely calibrated sensors. This includes both the intrinsic calibration of each sensor as well as the extrinsic calibration between all sensors. To illustrate the importance of sensor calibration, we show in Fig. 1 a sample from our automotive dataset, collected by a vehicle equipped with a camera, a lidar and a radar. The camera outputs 2D images, the lidar provides sparse 3D point clouds, and the radar yields sparse 4D point clouds (where the 4th dimension is expressed by pointwise Doppler values). The measurements from the different sensors are projected onto common reference frames for qualitative assessment of their alignment. Figure 1a–c show front views and a top view of the sensor measurements, respectively. Each view includes the uncalibrated state on the left and the calibrated state, resulting from our proposed approach, on the right. In Fig. 1a,b the uncalibrated and calibrated states of the lidar and radar with respect to the camera are shown; on the left there is a clear misalignment between the projected lidar points (colored points) and the scene, as well as between the radar dynamic track detections (cyan ‘+’ markers) and the moving vehicles. On the right-hand sides, the sensors are well calibrated using our method. In Fig. 1c, a bird’s-eye view of the uncalibrated and calibrated lidar (colored points) and radar (black points) is given; on the right the alignment correctness is discernible from the overlapping detections of objects such as vehicles and the right guard rail, compared to the considerable misalignment visible on the left.

Current calibration methods are usually categorized as either target-based or target-less.

### Target-based calibration

Target-based calibration is performed in a controlled environment. i.e., certain perceivable objects are known (pose, shape, and size) and the scene is usually static. The specific targets are used to obtain simultaneous measurements from multiple sensors that can be registered using geometric alignment. The result is the calibrated extrinsic and/or intrinsic parameters. The controlled targets are designed according to the intended sensor types in such a way that target measurements are clear and thus registration is more precise. e.g., planar surfaces with a distinct pattern (e.g., checkered) for cameras^11–14^, planar surfaces for lidars^11–18^ and trihedral corner reflectors for radars^16–19^.

Although the manner and environment in which they are conducted makes target-based methods highly accurate, they are also the reason why such techniques are not practical for everyday use. Sensors mounted to autonomous outdoor platforms are subject to vibrations and weather changes, can be mishandled (e.g., improper installment or calibration) or suffer impacts. Having their recalibration done manually by an expert, using dedicated equipment, is simply not a viable or scalable solution. Therefore, it is paramount that autonomous systems possess the ability to recover from an uncalibrated state online and automatically.

### Targetless calibration

Targetless methods are inherently suited for online use. Instead of using specialized apparatuses, the calibration is conducted based on perceived environmental features from natural scenes. For example, in the case of autonomous vehicles this would mean urban roads and highways. As such, the calibrated state is a state of maximal cross-sensor alignment of a set of some distinctive matching features. This type of calibration can therefore be regarded as two subsequent problems. The first is meaningful data extraction and interpretation, and the second is finding the best alignment based on those features.

When calibrating multiple instances of the same sensing modality (e.g., multiple cameras), cross-sensor alignment can usually be defined straightforwardly in terms of the correspondence between the sensor measurements (e.g., pixelwise correspondence between images in terms of RGB values, edges, etc.). However, when calibrating different sensing modalities such correspondences may not be so simple to define, since the features captured by each modality and their distinctiveness may vary. This makes targetless calibration a challenging task in both implementation and in achieving accuracies comparable to those attained by target-based methods.

## Related work

Existing targetless approaches, pertaining to extrinsic calibration, can be categorized based on the use of geometric features, odometry and object tracking, physical properties of different modalities, or deep-learning-based semantics matching.

### Geometry-based calibration

Geometric approaches usually rely on matching distinctive spatial features between the different modalities. Several methods suggested aligning distinct line features and edges to extrinsically calibrate a camera and lidar^20–23^. The calibration task was formulated as a registration problem between multiple planar regions, which are visible to both sensors and demonstrated how the camera intrinsic calibration can be included^23^. However, the solution required that the regions be associated a priori. Following a different approach, after applying structure from motion on a series of consecutive images, 3D registration of a lidar point cloud and a camera-generated point cloud resulted in the camera–lidar extrinsic calibration^24^. A method for calibrating multiple lidars and radars in an automotive setup used the lidars to construct a 3D reference map of the environment, to which the radar detections were then registered, thereby obtaining the relative sensor poses^25^.

### Odometry-and-tracking-based calibration

Odometry-based methods use ego-motion estimations for each sensor to extrapolate the rigid body transformations between them. Such approaches were applied to various sensor setups such as camera and lidar^26^, stereo cameras and lidar^27^ and camera and radar^28^. Object tracking has enabled sensor calibration by aligning one or more track traces to discern the relative poses between the sensors^29^.

### Physics-based calibration

Physical attributes of the camera–lidar duo were utilized to define criteria for measurement correspondence, where the predominant approach in these works was the correlation between lidar reflectance and image intensity^26,30–32^. Another technique attempted to match estimated local normals to the lidar mesh with the image intensity^33^.

### Deep-learning-based calibration

Deep neural networks (DNNs) have been used in different ways for extrinsically calibrating sensors of different types. Early approaches employed DNNs to assist in extracting features for use within an optimization network. Some used semantic information from camera and lidar to define a mutual-information-based objective function^34,35^. Others used a DNN to segment certain semantics in the image that could either be heuristically matched to detections from another sensor^36,37^ or to filter out certain objects^24^. RegNet^38^, CalibNet^39^, CalibRCNN^40^, RGGNet^41^ are examples of DNNs used for end-to-end calibration of a camera–lidar setup. Whether explicitly or implicitly, the feature extraction and matching are incorporated into the networks and the calibration parameters are regressed. Using a slightly different approach with respect to the output, LCCNet^42^, CFNet^43^ and DXQ-Net^44^ were trained to output a measure of the transformation correction needed for the given input. LCCNet did this directly, whereas CFNet and DXQ-Net provided this in a pixelwise manner over the projected lidar point cloud, termed calibration flow. Ultimately, all these methods relied on some form of ground-truth data for training, which is a major drawback for scalable and repeatable deployment in a commercial application. Conversely, self-supervised learning does not require any data labeling, making it especially suitable in situations where ground-truth annotations are not readily available and too costly to produce.

### Self-supervised learning

Self-supervised learning is a training method where one part of the signal is used to predict another part of the signal, thereby exposing underlying representations within the data. For example, this method was used for super-resolving a radar array^45^, up-sampling a camera frame^46–49^ or lidar measurements^50–52^.

Recent studies aimed to reformulate problems which were previously solved using supervised learning to enable the use of self-supervision instead. A prominent example of this are recent solutions for generating image semantics, such as object detection^53–55^ and semantic segmentation^56–58^, although, currently supervised methods outperform them. Self-supervision has also been used in different applications involving sensor measurement registration^59–61^. It has also been applied to intrinsic calibration of a monocular camera using information from subsequent images^62^.

The targetless solutions mentioned so far only perform calibration between two sensor types. When considering the desired redundancy in sensing modalities for safety–critical applications, such as autonomous vehicles, these approaches fall short of a more comprehensive solution.

Previous work on methods for calibrating three types of sensors is scarce. An online, targetless methodology for extrinsic multi-sensor calibration was outlined and demonstrated on a camera–lidar–radar setup^63^. Track-to-track association between sensor pairs was used for both detecting cross-sensor miscalibration and performing recalibration when necessary. One sensor was selected as a common reference, to which all other sensors were calibrated. The transformation between any two sensors could then be derived from their respective transformations to the reference sensor. This methodology does not take advantage of relevant cross-sensor correspondences making it more vulnerable to different environmental conditions. Meaning, choosing the camera as the reference sensor would cause the entire calibration process to fail under poor lighting. Furthermore, the reliance on dynamic object tracks exclusively means that this approach cannot be applied unless at least one such object exists and is being tracked by all the sensors.

In this work we propose a joint multi-sensor extrinsic calibration approach which consists of deducing the correct relative alignments between the sensors from several features across their respective signal measurements. The features are extracted and based on known physical properties of the different sensing modalities and modality-independent semantics. We propose two different methods to solve the calibration problem. The first consists of solving an optimization problem under a set of pairwise and global self-consistency constraints. The second approach is to train a DNN in a self-supervised manner without any ground truth, on the same minimization problem by using the objective function as the training loss. Once the DNN is trained, it is capable of single frame operation, making it robust to changes in environmental and operational conditions.

The remainder of the paper is arranged as follows: The next section outlines the proposed optimization-based and SSL-based calibration methods. Qualitative and quantitative experimental results of these methods are then presented. The subsequent section includes a discussion, followed by concluding remarks.

## Methods

A core concept of our proposed methods is to combine prior knowledge on the physical properties of different sensing modalities together with semantic information. The combination of the two is then used as constraints for cross-sensor alignment.

In this work we propose a holistic approach to the calibration problem in real-world conditions. We present two different calibration methods, the first is optimization-based and the second is SSL-based. Since these two are fundamentally different, the system-level architectures required for their deployment in a real-world setting vary significantly.

**Figure 2:**
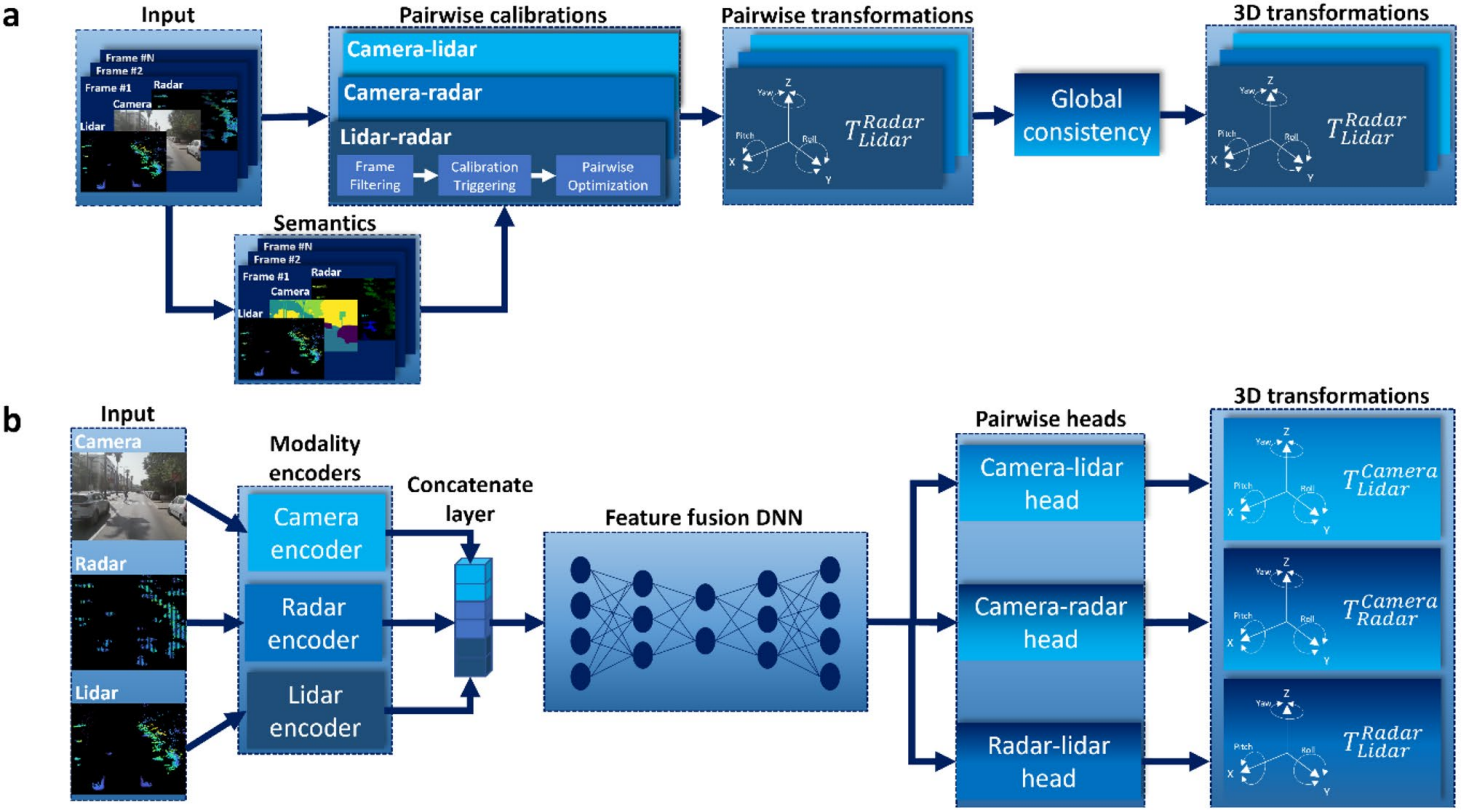
Block diagrams of the calibration methods. (**a**) Optimization-based method. The input to the system is a set of frames composed of time-synced data from all three sensors (camera, lidar, radar). Semantic features are extracted from all frames, which are passed along with the original frames to a three-stage pairwise calibration pipeline. The pipeline filters the frames, uses the selected frames to detect miscalibration and perform pairwise optimization. Finally, the obtained pairwise transformations are optimized with respect to a global consistency measure to obtain the refined 3D transformations. (**b**) SSL-based method. The input type is the same as in the optimization-based method, except in this case only a single frame is needed. The network encodes sensor data per modality, concatenates the encoded features and feeds them into a feature fusion network. Its output is split and passed through three task heads, one for each sensor pair, to generate the required pairwise transformations.

The optimization-based calibration process included logics for continuous frame aggregation, recalibration triggers and calibration procedure, as illustrated in Fig. 2a. The input to the system is a set of frames composed of pairwise, time-synced data from all 3 sensors (camera, lidar and radar). Semantic features are extracted from all frames, which are passed along with the original frames to a three-stage pairwise calibration pipeline. The pipeline filters the frames and uses the selected frames to detect miscalibration and perform pairwise optimization. Finally, the obtained pairwise transformations are optimized with respect to a global consistency measure to obtain the refined 3D transformations.

The model used is illustrated in Fig. 2b and consists of three sensor-wise encoders, a global fusion module and separate pairwise heads. The three encoders extract separate features for each modality, which can be implemented as convolutional layers, transformers, or multi-layer perceptrons. These features are then concatenated and fed into a global fusion module, which regresses the transformation parameters between all sensor pairs. The information then flows to three pairwise heads each outputting the corresponding 6 degrees of freedom represented by three Euler angles and three translation components in 3D cartesian space. Similarly, to the encoders, the fusion module and the pairwise heads can be implemented using a variety of flavors. In our implementation we chose a convolutional approach for simplicity.

The SSL-based method is fundamentally different from the optimization-based method in several aspects affecting its design and deployment. During inference the DNN does not require any additional semantic information and can be applied on a single-frame basis. As a result, there is no need for real time sample collection or triggering mechanisms, making the overall system design much simpler and robust.

### Physics and semantics informed calibration

#### Feature extraction

Camera-semantics were extracted in the form of object detection and semantic segmentation using YOLOR^66^ and SegFormer^67^ respectively. Lidar point clouds underwent uniform sampling, which was previously found to benefit different feature extraction algorithms^68^ and were then clustered using DBSCAN^69^ to extract geometric objects in the scene. Lidar semantics utilized RangeNet++^70^ and RANSAC^71^ successively to segment the drivable area. Radar data underwent clustering and tracking to distinguish moving objects. In addition, a weakly-supervised method^72^ for creating a radar-based DNN was used to segment the drivable area.

#### Pairwise constraints

Three pairwise feature-based constraints were devised for each sensor pair that were a combination of physical properties of the sensing modalities and semantic information. These were later represented by smooth loss functions to be minimized in each of the methods.

With respect to the camera–lidar pairing, the projected lidar clusters are expected to correspond to relevant segmented objects in the image which are known to reflect lidar signals, such as: vehicles, pedestrians, signs, poles, buildings, fences, and vegetation. This was expressed as a loss term equal to the summed distances between the clusters and the segmented objects. As the sky is a known non-reflective region, lidar detections projected on regions in the image which were segmented as ‘sky’ or outside of the FOV were given a penalty.

The camera–radar pair utilized one of the radar’s strong suites, which is the ability to measure Doppler. Since radar-based tracked clusters were necessarily associated with dynamic road users (vehicles, pedestrians, etc.), the associated loss was based on a sum of the distances between each projected radar-based tracked cluster and its nearest detected road user’s center in the image. In addition, similarly to the camera–lidar projection, radar detections should not be projected on regions in the image which were segmented as ‘sky’ or outside of the camera FOV, hence any such occurrences were given a penalty.

The lidar–radar correspondence was reflected by the alignment between the projected lidar-based ‘drivable area’ and the radar-based ‘drivable area’ in the frame, measured by their intersection-over-union (IOU) value. In addition, since objects clustered in the lidar point cloud included dynamic road users, radar-based tracked clusters were expected to align with certain lidar clusters. The respective loss was defined as the sum of the distances between each projected radar-based tracked cluster and its nearest object cluster center in the lidar point cloud.

#### Global constraints

Under the assumption that the sensors are intrinsically calibrated, the transformations, now an expression of the extrinsic parameters alone, become linear and can be expressed in the $\left[R|t\right]$ matrix form of ${\mathbf{T}}_{i}^{j}=\left[{\mathbf{R}}_{i}^{j} {\mathbf{t}}_{i}^{j}; {0}_{1\times 3} 1\right]$ where ${\mathbf{R}}_{i}^{j}$ and ${\mathbf{t}}_{i}^{j}$ are, respectively, the $3\times 3$ rotation matrix and $3\times 1$ translation vector from reference frame $i$ to $j$. By creating a closed-loop cyclic transformation, the global consistency constraint takes on the form of ${\mathbf{T}}_{lidar}^{camera}\cdot {\mathbf{T}}_{radar}^{lidar}{\cdot \mathbf{T}}_{camera}^{radar}={\mathbf{I}}_{4\times 4}$ with ${\mathbf{I}}_{4\times 4}$ being a $4\times 4$ identity matrix. For example, a point starting at some pixel on the camera, and undergoes transformation to the lidar, then the radar and back to camera, should return to its original pixel location. Similarly, with a lidar point or radar detection. In our implementation, as with the pairwise conditions, we regarded this constraint as an additional loss term to be minimized. We chose to express this loss as an explicit function of the cyclic transform’s Euler angles and translation vector, equal to the ${l}_{1}$-norm of these elements.

### Optimization-based method details

#### Frame aggregation and filtering

Samples corresponding to measurements from sensor pairs are collected continuously throughout the drive. Each of the two modalities’ measurements undergoes filtering to determine whether the frame is suitable for calibration, and if so, both measurements are processed further to obtain distinctive features to be used in the calibration process of the respective modalities. A unique set of requirements for a frame to be valid pertains to each modality pair: camera–lidar, camera–radar and lidar–radar. These criteria referred to the physics, semantics, and geometric conditions. To be utilized in the calibration stage and were empirically found to encourage the numerical stability of the optimization process.

The conditions found in this study were occurrences of the semantic regions of ‘sky’ and ‘drivable area’ in the camera image of at least 20% and 10% respectively. In addition, a requirement of at least three instances of road users was also included. Lastly, we required the radar tracker to report at least three dynamic clusters. In all cases a time sync of under 5 ms between measurement pairs was required to alleviate temporal influence.

#### Re-calibration triggers

The conditions for triggering the optimization-based re-calibration process are checked periodically with respect to the current set of gathered frames. These consist of three metrics, corresponding to each of the modality pairs. Essentially, re-calibration between two sensing modalities is triggered when a significant misalignment between their measurements is detected. This is done based on the perceived association between different objects and/or regions identified in each of the sensors’ measurements.

Misalignment for the camera–lidar pair is measured by summation of the number of projected lidar points that are projected outside of their associated segmentation in the image. Re-calibration is triggered if that sum exceeds 1% of the entire point cloud.

Similarly, misalignment for the camera–radar pair is measured by summation of the number of projected radar detections that fall outside of their associated segmentation in the image. Re-calibration is triggered if the number of dynamic detections outside of their associated object segmentation in the image is greater than one.

Lastly, misalignment for the lidar–radar pair is measured by summation of the number of projected radar-based dynamic detections that fall outside of their associated objects in the lidar point cloud and by comparing semantic regions. Re-calibration is triggered if the number of dynamic detections projected outside of their respective lidar-based bounding boxes is greater than one. Re-calibration could also be triggered if the percentage of lidar points is greater than 1% for those which are classified as ‘drivable area’ and are projected outside of the radar-based ‘drivable area’.

#### Optimization process

We formulated the calibration task as an optimization problem in which the following objective is to be minimized:

1 $${\mathop{{\text{min}}}\limits_T} L\left({L}_{P}\left(T, \varphi \right),{L}_{G}\left(T\right)\right)$$

with

2 $$L\left({L}_{P}\left(T, \varphi \right),{L}_{G}\left(T\right)\right)=\left[{1+L}_{P}\left(T, \varphi \right)\right]\left[1+{L}_{G}\left(T\right)\right]$$

where ${L}_{P}$ and ${L}_{G}$ are the pairwise feature-based loss and global self-consistency loss respectively. $T$ is the set of all pairwise rigid body transformations and $\varphi$ is the set of all features across a given collection of frames for all modalities pairs.

The optimization was run with respect to triggered pairs of modalities as well as the entire sensor suite. The loss was accumulated only over samples from uncalibrated sensor couples with the addition of the global self-consistency term. The solution was obtained iteratively using the sample-based Differential Evolution^73^ and Nelder-Mead^74^ algorithms, consecutively.

### SSL-based method details

#### Training methodology

The SSL-based method utilizes end-to-end training where the multi-modal DNN takes as input a trio of uncalibrated RGB image, lidar point cloud and radar point cloud and outputs a set of 3D transformation matrices for each of the sensing modalities pairs. The DNN is trained in a self-supervised manner and no labels or ground truth information is used.

**Figure 3:**
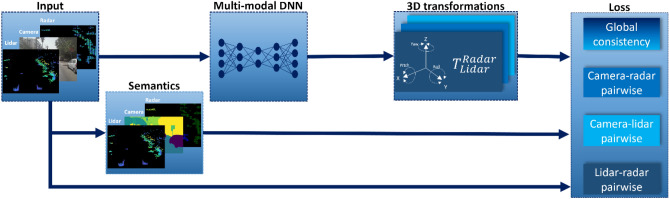
SSL training scheme. Each frame is passed on to a multi-modal DNN which simultaneously predicts 3D transformations for all sensor pairs, and to pretrained sensor specific DNNs (whose parameters are frozen during training) which generate scene semantics. The output of all DNNs, along with the original frame, are all used to compute the various losses (pairwise losses and global self-consistency loss).

During training, the output of the DNN, together with semantic information extracted from the camera–lidar–radar trio and the measurements themselves are used to calculate the pairwise losses as well as the global self-consistency loss as illustrated in Fig. 3.

The training dataset consists of multiple data-collection sequences, each with different sensor alignment making the dataset varied and more challenging. To augment the variability and improve the generalization of our SSL-based method, we randomly rotated and translated the lidar and radar point clouds during training to simulate a much larger set of uncalibrated situations.

#### Loss function

The loss function for the SSL-based method is a sum of the pairwise and global self-consistency loss:

3 $$L= {L}_{P}\left({T}_{\theta }\left(x\right),{\varphi }_{x}\right)+ {L}_{G}\left({T}_{\theta }\left(x\right)\right)$$

With ${T}_{\theta }(x)$ being the set of all pairwise rigid body transformations learned by the DNN which is parametrized by the weights $\theta$ on frame $x$; ${\varphi }_{x}$ is the set of all precomputed semantic and physical features for all sensing modalities of frame $x$.

#### Implementation details

Training was implemented in PyTorch, the optimizer used was Adam with ${\beta }_{1}=0.9$, ${\beta }_{2}=0.999$, batch size was 4, and learning rate used cosine decay from $3\times {10}^{-4}$ to $3\times {10}^{-7}$. All configurations were trained until convergence on a single A6000 GPU which took about 20 epochs.

## Experimental setup

To demonstrate and evaluate our approaches, we used a sensor setup which included a camera, lidar and radar in an automotive system setting. The data was collected in different, uncontrolled urban and highway environments over multiple days, where the sensors were mounted at the beginning of each day and removed at the end of each day. Meaning, sensor alignment varied across locations and days. The dataset contained 240,000 frames, where each frame contains temporally synced measurements from the camera, lidar and radar. The data was split to 200,000 for training and 40,000 for validation. The data for the validation was taken from different days and locations to avoid the appearance of similar frames in both datasets, which could have occurred in the case of simple random split. An example frame from the collected dataset is provided in Fig. 1.

The camera included in the setup (Leopard Imaging LI-OV10650-495-GMSL2-120H) had a field of view (FOV) of $116^\circ H\times 58.5^\circ V$ that captures $1824\times 940$ resolution images. The lidar (Cepton Vista-P60) had a FOV of $60^\circ H\times 24^\circ V$ and $0.25^\circ H\times 0.25^\circ V$ angular resolution. The radar—an experimental collocated multiple-in multiple-out (MIMO), frequency-modulated continuous wave (FMCW) radar—was configured to have a FOV matching that of the lidar and had an angular resolution of $1.6^\circ H\times 6^\circ V$. Both the lidar and the radar were configured to a minimum range of 5 m and maximum range of 80 m.

An additional dataset for quantitative comparison was collected under controlled, stationary conditions. The controlled setup included three trihedral reflective corners placed in a leveled empty lot at varying locations to allow for spatially diverse error measurement. These targets were recorded for 150 frames by each of the sensors and their positions were manually pinpointed within each modality’s coordinate system. These recordings took place adjacent to the data collection for the validation set, thus ensuring similar calibration conditions.

**Figure 4:**
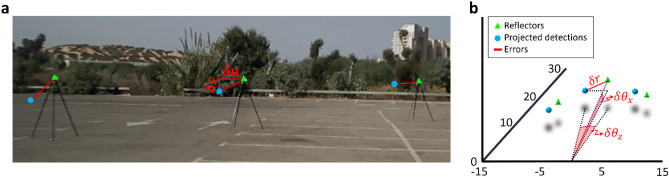
Controlled test setup. The controlled test setup was made up of three trihedral corner reflectors (green triangles) at varying range-azimuth combinations to allow for spatially diverse error measurement. Simulated projections (blue circles) were added to illustrate error measurement (red lines) expressed in azimuth ($\delta {\theta }_{z}$), elevation ($\delta {\theta }_{x}$), and range ($\delta r$). (**a**) Controlled test setup from the camera’s perspective, where initially the angular errors were measured pixelwise horizontally ($\delta u$) and vertically ($\delta v$). (**b**) Isometric view of the controlled test setup; Cartesian axes’ units are in meters.

An example frame from the controlled test setup is provided in Fig. 4. A view of the controlled test setup from the camera’s perspective is shown in Fig. 4a. Calibration errors of the camera-paired transformations (in red) were extracted from images in terms of horizontal and vertical pixelwise deviations ($\delta u$ and $\delta v$, respectively). These were then converted to 2D angular errors of azimuth and elevation. An isometric view of the controlled test setup is shown in Fig. 4b, illustrating the lidar–radar 3D calibration errors (in red) which were measured in terms of azimuth ($\delta {\theta }_{z}$), elevation ($\delta {\theta }_{x}$), and range ($\delta r$). The camera–lidar–radar inconsistency was expressed in the same way.

## Results

**Figure 5:**
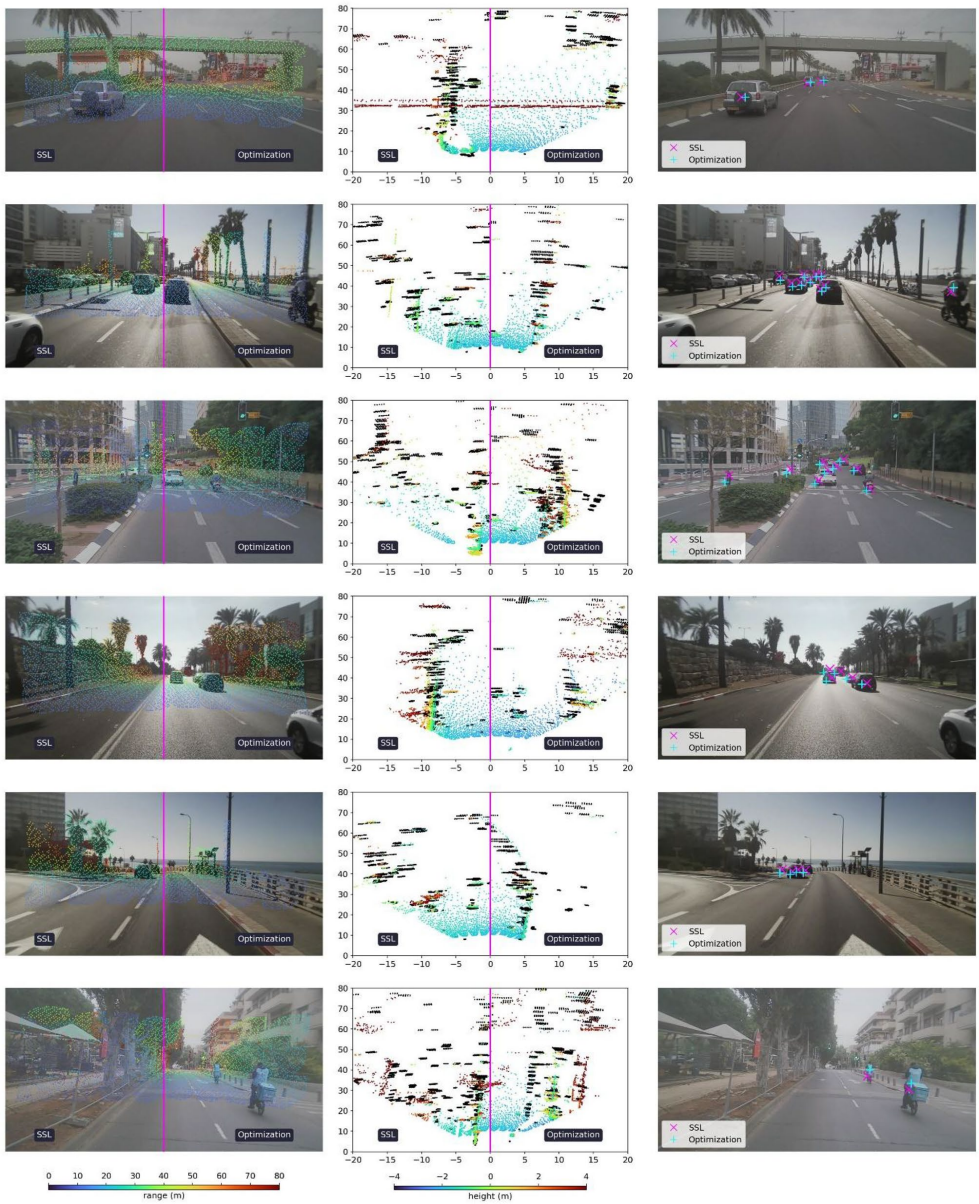
Sample results from the validation dataset. From left to right: Calibration results of the optimization-based and SSL-based methods for camera–lidar, lidar–radar and camera–radar, respectively. On the left column, the camera–lidar calibration shows the projected lidar point cloud onto its corresponding image, with colormap representing range in meters. In the middle column, the lidar–radar calibration is portrayed in bird’s eye view. The lidar is represented in a colormap for height in meters, whereas the radar detections are represented as black points. For both pairs (camera-lidar and lidar-radar), each frame is split along the middle, where the optimization-based calibration is displayed in the right half and the SSL-based calibration in the left half. On the right column, center of mass from tracked radar clusters are projected onto the corresponding images using the calibrations from both proposed methods.

Qualitative assessment of our proposed methods can be done by projecting one sensor’s measurements onto another sensor’s frame of reference. Sample results from the validation dataset for both the optimization-based and SSL-based configurations are provided in Fig. 5, where the results show good correspondence between the sensor measurements. From left to right, the camera–lidar alignment is shown in the image plane where the colormap used for the lidar point cloud represents the range in meters. The top view plot displays the lidar–radar correspondence, where the colormap is used to represent the lidar points’ height in meters, whereas the radar detections are displayed in black to better distinguish between the two sensing modalities. To illustrate the camera–radar calibration, we used the centers of mass from tracked radar clusters, as it makes it easier for the reader to visualize the association of the dynamic objects in the camera–radar pair.

For both pairs (camera–lidar and lidar–radar), each frame is split along the middle, where the optimization-based calibration is displayed in the right half and the SSL-based calibration in the left half. On the right column, center of mass from tracked radar clusters are projected onto the corresponding images using the calibrations from both proposed methods.

Quantitative evaluations were carried out using the controlled test setup. The methodology consisted of deriving all three pairwise calibrations from the validation dataset, then applying the different transformation matrices to the controlled test measurements and calculating the resulting alignment errors.

As a baseline, we compared our proposed optimization-based and SSL-based methods to the object-tracking-based multi-sensor calibration^63^. Due to lack of available sources, we used our implementation of this method and replaced the original camera-based 3D object detection^64^ with a stronger detector^65^. The errors reported in Table 1 are an average over the validation dataset and all three targets in the controlled test setup.

**Table 1:** Quantitative evaluation of the proposed methods.

	Pairwise calibration errors									Global calibration errors		
	Camera–Lidar			Camera–Radar			Lidar–Radar			Camera–Lidar–Radar		
	Az [deg]	El [deg]	R [m]	Az [deg]	El [deg]	R [m]	Az [deg]	El [deg]	R [m]	Az [deg]	El [deg]	R [m]
Object-tracking-based calibration^63^	0.55	2.01	–	1.92	3.13	–	1.46	1.87	0.22	–	–	–
Pairwise optimization	0.21	0.28		0.20	0.44		0.97	**0.21**	0.14	1.43	0.83	0.01
Pairwise SSL	**0.07**	**0.09**		1.36	0.29		1.17	1.09	0.30	0.14	0.61	0.01
Joint optimization	0.22	0.27		0.20	0.45		**0.36**	0.72	0.14	0.09	**0.01**	0.01
Joint SSL	0.08	0.20		**0.02**	**0.14**		0.51	0.61	**0.13**	**0.02**	0.02	**0.01**

Average calibration errors, computed with respect to three reflective corners, are provided for all modality pairs as well as for the closed transformation loop between all sensors. The smallest obtained errors appear in bold, and the second-best results are underlined. Our optimization method was run once without the self-consistency loss in a decoupled manner (pairwise optimization) and once with the self-consistency loss in a coupled manner (joint optimization). The SSL method was implemented once as three decoupled networks (pairwise SSL), and as a single network trained with the self-consistency loss (joint SSL). The calibration errors obtained by the joint optimization and SSL methods demonstrate the advantage of introducing the self-consistency constraints. Overall, the joint SSL outperforms all other methods, being the only one that consistently produces either the best or second-best results.

In addition, a quantitative evaluation was also carried out for a closed-loop transformation to gauge the global self-consistency. The lidar was chosen as the reference sensor as it facilitated a 3D error representation. Accordingly, the closed-loop transform was the result of a composition of all pairwise transformations in a cyclic order beginning and ending with the lidar reference frame.

To evaluate the significance of including self-consistency considerations as part of the solution, we examined our proposed optimization-based and SSL-based methods with and without the self-consistency constraint. Under the non-consistent optimization-based configuration the optimization process was applied to each sensor pair separately using its corresponding objective function, yielding three independent pairwise calibrations. The pairwise SSL configuration was carried out by training three separate DNNs, each with a different pairwise loss function, to regress the corresponding sensors’ calibration parameters solely from their respective measurements. The joint optimization consisted of performing the pairwise optimization which was then refined by a simultaneous joint optimization of all three sensors with respect to all pairwise objectives as well as a self-consistency constraint. Meanwhile, the joint SSL configuration included a single DNN trained to predict all three sensors’ pairwise calibrations given their measurements with the loss function being a composition of the pairwise losses and the self-consistency requirement. Additional details concerning the objective and loss functions can be found in the Methods section. The performances of each of these configurations are also reported in Table 1 highlighting the advantage of including the self-consistency constraints.

Additionally, we examined the joint SSL-based method’s capacity for calibration under abrupt and large changes. This examination simulates real-world conditions where a sensor might get miss-aligned for various reasons, such as vibrations, impact, material fatigue and more. In each scene the sensors were given arbitrary initial alignment errors and the DNN was used to infer the correct alignments from that scene alone.

**Figure 6:**
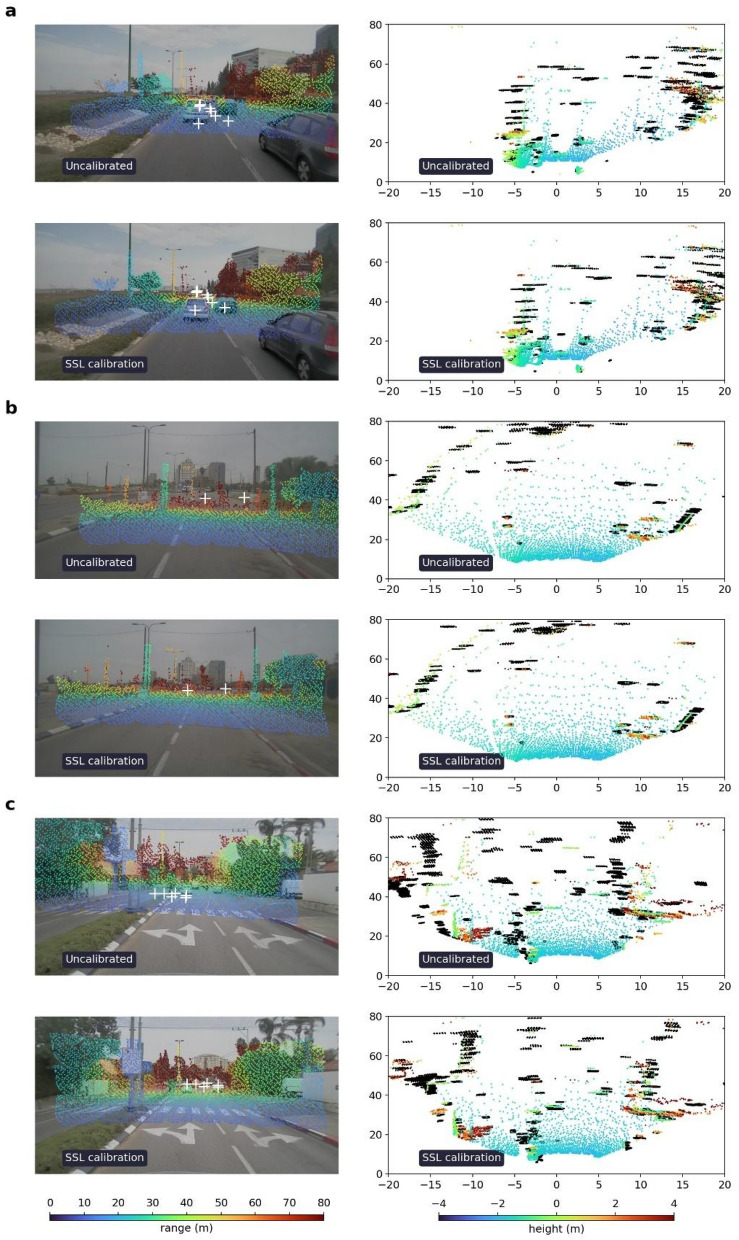
Robustness to abrupt changes. (**a**–**c**) Examples of the joint SSL-based method in different scenes and various initial un-calibrated conditions. Sensor spatial measurements; x and y axes in meters. Each sample frame shows the uncalibrated on the top row and SSL-based calibrated on the bottom row. On the left column, a lidar point cloud is projected on top of a camera image with a colormap representing range in meters. In addition, the center of mass of tracked radar clusters are shown using white ‘+’ markers. On the right column, top view of the lidar point cloud with colormap representing height in meters. Radar detections are shown as black points.

Qualitative results of this evaluation are shown in Fig. 6. The camera-paired uncalibrated and calibrated states are shown on the left with the projection of the lidar and radar measurements onto the corresponding image. On the right, a bird’s-eye-view of the lidar–radar uncalibrated and calibrated states in each scene is shown in the lidar’s coordinate frame. The results show good correspondence when applying our proposed SSL-based method and its robustness and generalization ability.

**Figure 7:**
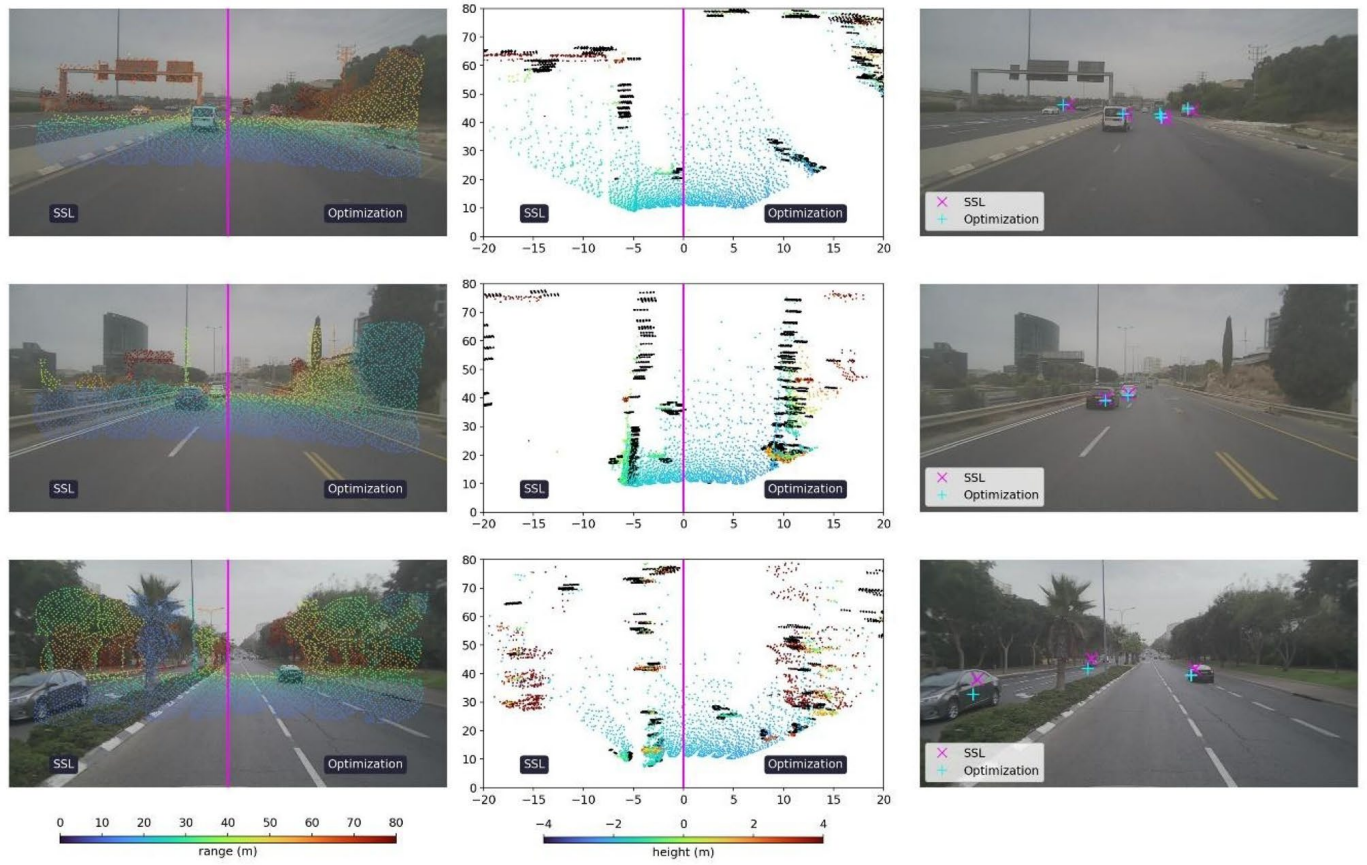
Sample frames in highway environment. From left to right: Calibration results of the optimization-based and SSL-based methods for camera–lidar, lidar–radar and camera–radar, respectively. On the left column, the camera–lidar calibration showing the projected lidar point cloud onto its corresponding image, with colormap representing range in meters. In the middle column, the lidar–radar calibration is portrayed in bird’s eye view. The lidar is represented in a colormap for height in meters, whereas the radar detections are represented as black points. For both pairs (camera–lidar and lidar–radar), each frame is split along the middle, where the optimization-based calibration is displayed in the right half and the SSL-based calibration in the left half. On the right column, center of mass from tracked radar clusters are projected onto the corresponding images using the calibrations from both proposed methods.

**Figure 8:**
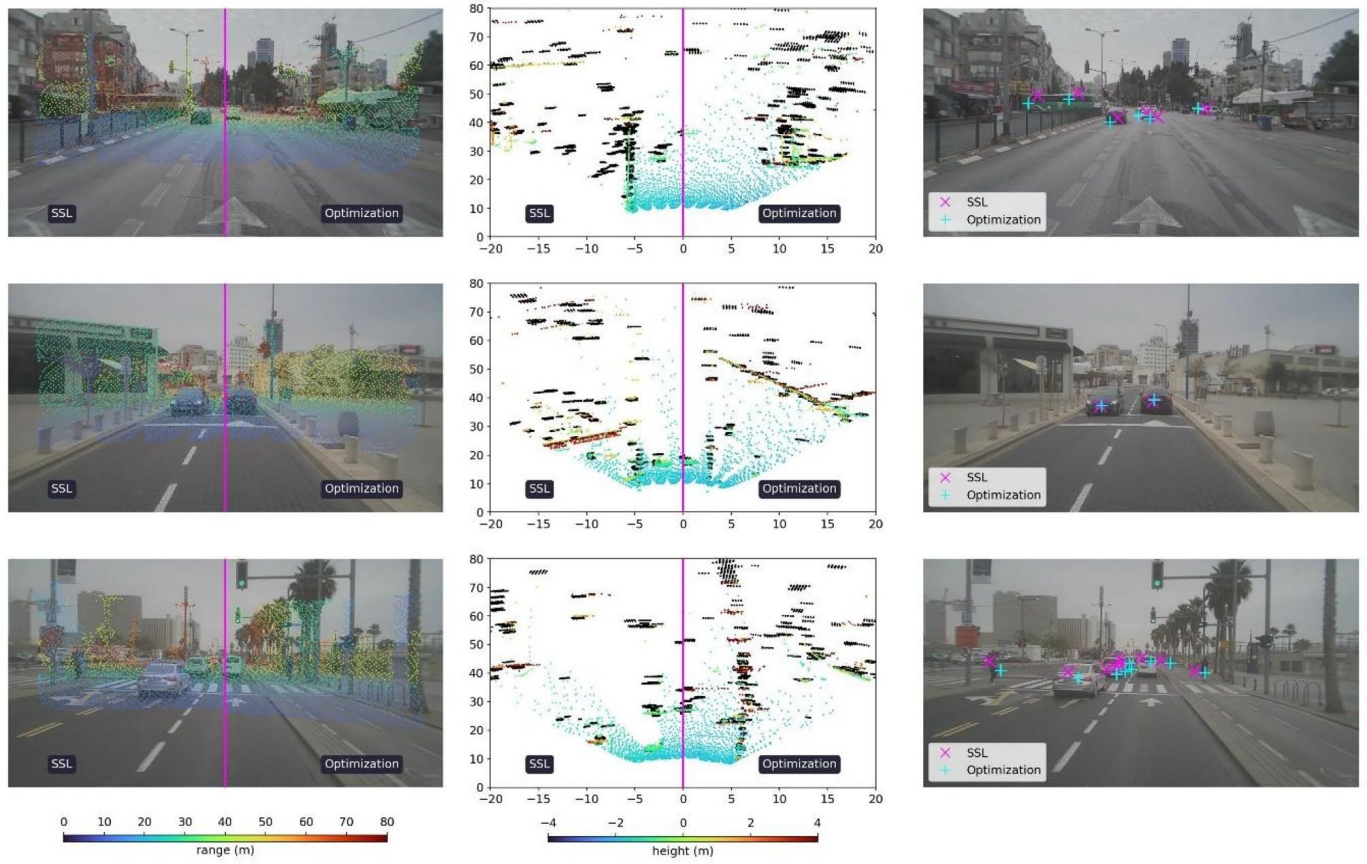
Sample frames in urban environment. From left to right: Calibration results of the optimization-based and SSL-based methods for camera–lidar, lidar–radar and camera–radar, respectively. In the left column, the camera–lidar calibration shows the projected lidar point cloud onto its corresponding image, with colormap representing range in meters. In the middle column, the lidar–radar calibration is portrayed in bird’s eye view. The lidar is represented in a colormap for height in meters, whereas the radar detections are represented as black points. For both pairs (camera–lidar and lidar–radar), each frame is split along the middle, where the optimization-based calibration is displayed in the right half and the SSL-based calibration in the left half. On the right column, centers of tracked radar clusters are projected onto the corresponding images using the calibrations from both proposed methods.

We also examined the sensitivity of the proposed optimization-based and SSL-based methods with respect to different driving environments, mainly urban and highway scenarios. The results are provided in Figs. 7 and 8 showing advantage for the SSL-based method over the optimization-based method. Furthermore, the optimization-based method performs slightly better in urban conditions than in highway conditions, whereas the SSL-based method shows similar qualitative performance in both environments.

A quantitative examination of the two driving environments is provided in Table 2 confirming the qualitative results. There we see that the optimization-based method performs better in urban environments than in highway environments. In contrast, the SSL-based method shows robustness to these changes with similar performance metrics.

**Table 2:** Quantitative evaluation of the effects of driving environments.

	Pairwise calibration errors									Global calibration errors		
	Camera–Lidar			Camera–Radar			Lidar–Radar			Camera–Lidar–Radar		
	Az [deg]	El [deg]	R [m]	Az [deg]	El [deg]	R [m]	Az [deg]	El [deg]	R [m]	Az [deg]	El [deg]	R [m]
Highway												
Joint optimization	0.42	0.65	–	0.08	0.60	–	0.31	1.17	0.13	0.17	0.02	0.01
Joint SSL	**0.01**	0.22		0.09	**0.08**		0.49	0.66	0.14	0.06	0.03	0.07
Urban												
Joint optimization	0.13	0.21	–	0.13	0.49	–	**0.02**	0.69	0.13	0.06	**0.01**	**0.01**
Joint SSL	0.10	**0.18**		**0.03**	0.37		0.51	**0.61**	**0.13**	**0.03**	0.04	0.04

Average calibration errors, computed with respect to three reflective corners, are provided for all modality pairs as well as for the closed transformation loop between all sensors. The optimization-based method performs better in urban environment than in highway environment. The SSL-based methods show robustness to these changes with similar performance metrics. The smallest obtained errors appear in bold, and the second-best results are underlined.

## Discussion

To achieve high reliability and meet strict safety standards during real-world conditions, redundancy in sensing modalities is usually required. This requirement, coupled with typical operational conditions, brings about the need for frequent multi-sensor calibrations. Methods requiring specialized equipment and manual operation do not pose a viable and scalable solution, giving rise to the need for accurate, reliable, and automated methods capable of performing multi-sensor calibration in uncontrolled settings.

We take a holistic approach, also considering the system-level components required for scalable real-world deployment and propose two different approaches for calibration. The first is based on an optimization problem formulation of the calibration assignment, while the second is based on framing the task as a learning problem.

We base both proposed methods on a similar set of constraints that take account of the physical properties of the different signals as well as semantic information extracted from all sensing modalities, to produce pairwise alignment measures. Encapsulating these is the requirement that all cross-sensor transformations are globally self-consistent, meaning that a cyclic transformation should project data points to their original coordinates.

When viewing the calibration process as a component aimed for scalable and robust deployment there are considerable differences between the proposed optimization-based and SSL-based methods. The optimization-based method requires additional sub-components preceding the actual calibration in the form of procedures to aggregate appropriate samples for calibration and continuously querying some criteria for triggering re-calibration. These are important components which for the most part have previously been disregarded and are directly tied to the overall calibration performance. It is therefore our belief that they should be inseparable from the calibration method.

The SSL-based method requires a larger data collection effort prior to deployment in comparison to the optimization-based solution. Unlike most previous work, our proposed SSL-based method does not require any manual labeling or ground truth system. Instead, by using the set of constraints in the loss function during training, it is possible to train end-to-end in a self-supervised manner. Thus, greatly reducing the overall cost and resources required.

By visually comparing the two proposed methods, as shown in Fig. 5, it is evident that both methods can successfully solve the calibration task. Their desirability for use in specific applications is based on other system-level considerations, such as available computing resources during operation that might tip the scale in favor of the optimization-based method for example.

The results provided in Table 1 show that, by large, the SSL-based method achieves superior results to the optimization-based method. This implies that a DNN can potentially learn to extract and match features beyond the capability of its heuristics-based counterpart, allowing it to maintain its performance across varying scenes.

Additionally, we demonstrate the importance of imposing the global self-consistency constraint which yielded improved performance, as reflected by the overall lower error metrics. The results reported in Table 1 also suggest that apart from ensuring an overall self-consistent calibration, including the global constraint in the calibration process facilitates mutual corrective behavior between the different sensor pairs. Meaning, parameters derived reliably in one sensor pair can increase the reliability of parameters in other sensor pairs that now become coupled via the global constraint.

The optimization-based method includes components for sample aggregation and calibration triggering logics. The sample aggregation provides a variety of features across multiple frames, thereby increasing the optimization numerical stability. However, as the frame selection criteria refer to the presence or absence of certain features in the environment, in some cases the time required to gather enough samples might be significant. Similarly, calibration is triggered by misalignment measured with respect to particular features in the scene. If none can be found, then recalibration might be delayed.

In contrast, during deployment, the DNN can generate a calibration solution on a single frame basis. Meaning, there is no need for additional sample collection procedure or re-calibration triggering mechanism. This greatly simplifies the overall system design. In addition, this capability also makes the SSL-based method more robust and resilient to abrupt changes and sensor displacements that might occur in real-life settings due to vibrations, shock, temperature differences, and more. This is demonstrated in Fig. 6 where the differences between the uncalibrated and calibrated frames are clearly visible.

Driving environments and conditions are important considerations during real-world deployment. Since we aim to fulfill the desire for an automated, online calibration based solely on information gathered from the scene, we examined the effects of urban versus highway driving. As can be seen in Figs. 7 and 8, and quantitatively supported in Table 2, the SSL-method is more resilient to such changes with similar performance in both environments.

It is worth noting that achieving the complete calibration is predicated on the normal operation of all sensors. If one of the sensors is faulty or is rendered unusable due to environmental conditions (such as a standard camera at night or in heavy fog), its corresponding pairwise calibrations cannot be attained and the self-consistency cannot be measured.

## Conclusion

In this work we address the critical issue of online multi-sensor calibration in uncontrolled environments. To that end we consider a setup including the currently most prominent sensors for perception purposes: camera, lidar, and radar. We propose a physics and semantic informed methodology by which cross-sensor alignments are gauged based on matching semantics and conformation to known physical properties of the different sensing modalities.

We propose two fundamentally different approaches to the calibration problem: an optimization-based method and an SSL-based method. Under an optimization framework we provide a representative minimization problem in which a calculable measure of pairwise misalignments and global self-inconsistency is to be minimized. The second approach is realized in the form of a DNN trained in a self-supervised manner, meaning there is no reliance on any kind of ground-truth data. We demonstrate the merit of both methods on an automotive system setting, although their validity may extend to others as well.

The optimization-based method, empirically shown to require the stabilizing effect of running on multiple measurement frames, is accompanied by frame aggregation and filtering logics. Furthermore, considering real-world application, recalibration triggering is conceived and included as well. Conversely, the SSL-based solution can be self-contained, as a result of its demonstrated capacity for precise calibration from a single frame. Both solutions are shown to provide precise and robust joint calibration in a real-world setting, beyond the capabilities of current state-of-the-art methods. Experimental results support our selection of semantic features and characteristics of the different sensing modalities. Our inclusion of the self-consistency constraint in both approaches is shown to significantly improve the calibration results, as a facilitator of mutual corrective behavior between the different sensor pairs.

In a broader sense, this work illustrates how direct-optimization and self-supervised learning can be related, through a common optimization framework. We hope this work will inspire additional research in self-supervised learning and open new avenues traditionally only solved by optimization frameworks.

## Data Availability

The data generated to support the findings of this study are available from the corresponding author upon reasonable request and for non-commercial purposes only.
